# m6A RNA methylation-mediated NDUFA4 promotes cell proliferation and metabolism in gastric cancer

**DOI:** 10.1038/s41419-022-05132-w

**Published:** 2022-08-17

**Authors:** Weihong Xu, Yanan Lai, Yunqi Pan, Meiyu Tan, Yanyun Ma, Huiming Sheng, Jiucun Wang

**Affiliations:** 1 School of Life Sciences, Fudan University, Shanghai, 200433 China; 2 Department of Clinical Laboratory, Shanghai Tongren Hospital, Shanghai, 200336 China; 3 Department of Anthropology and Human Genetics, School of Life Sciences, Fudan University, Shanghai, 200438 China

**Keywords:** Gastric cancer, Cancer metabolism

## Abstract

Gastric cancer (GC) is a malignancy with poor prognosis. NDUFA4 is reported to correlate with the progression of GC. However, its underlying mechanism in GC is unknown. Our study was to reveal the pathogenic mechanism of NDUFA4 in GC. NDUFA4 expression was explored in single-cell and bulk RNA-seq data as well as GC tissue microarray. Mitochondrial respiration and glycolysis were estimated by oxygen consumption rate and extracellular acidification rate, respectively. The interaction between NDUFA4 and METTL3 was validated by RNA immunoprecipitation. Flow cytometry was used to estimate cell cycle, apoptosis and mitochondrial activities. NDUFA4 was highly expressed in GC and its high expression indicated a poor prognosis. The knockdown of NDUFA4 could reduce cell proliferation and inhibit tumor growth. Meanwhile, NDUFA4 could promote glycolytic and oxidative metabolism in GC cells, whereas the inhibition of glycolysis suppressed the proliferation and tumor growth of GC. Besides, NDUFA4 inhibited ROS level and promoted MMP level in GC cells, whereas the inhibition of mitochondrial fission could reverse NDUFA4-induced glycolytic and oxidative metabolism and tumor growth of GC. Additionally, METTL3 could increase the m6A level of NDUFA4 mRNA via the m6A reader IGF2BP1 to promote NDUFA4 expression in GC cells. Our study revealed that NDUFA4 was increased by m6A methylation and could promote GC development via enhancing cell glycolysis and mitochondrial fission. NDUFA4 was a potential target for GC treatment.

## Introduction

Gastric cancer (GC) is a malignancy ranked as the fourth occurrence of cancer [1]. Every year, more than 1 million GC cases are diagnosed and it causes hundreds of thousands of deaths. Current management of GC is composed of surgery, chemotherapy, radiotherapy, and immunotherapy. However, despite the great progress of cancer treatment, the 5-year surgival of GC patients is <30% [2]. Therefore, it is urgent to have a better understating of GC development.

Cancer is heterogeneous and various cancers use both glycolysis and oxidative phosphorylation for energy metabolism. The cell survival, proliferation, invasion, metastasis, and drug resistance of GC are energy-demanding processes, fuelled by glycolysis and oxidative metabolism [3]. Shift metabolism profile from mitochondrial oxidative phosphorylation to aerobic glycolysis is a key for tumor cell growth and metastasis [4]. As such, dysfunctional changes in cellular energy metabolism in GC warrant further investigation.

NADH dehydrogenase (ubiquinone) 1 alpha subcomplex 4 (NDUFA4), which encodes a subunit of the electron transport chain complex belonging to the respiratory chain of mitochondria to produce ATP [5]. Since tumor cells tend to evade apoptosis, the regulation of mitochondria-mediated cell death is commonly detected in different tumors [6]. This resistance of apoptosis is associated with mitochondrial dynamics, including fusion and fission, which are reported to closely correlate with cancer development [7]. NDUFA4 is reported to be differentially expressed in renal cell carcinoma and is associated with cancer-specific survival [8]. NDUFA4 could contribute to the growth and metastasis of human lung cancer cells through altering the transduction of the AKT and ERK pathways [9]. NDUFA4 promotes proliferation, reduces apoptosis and activates the oxidative phosphorylation pathway of GC cells [10]. Moreover, NDUFA4 facilitates glycolysis rather than oxidative phosphorylation in colorectal cancer cells by regulating genes involved in glycolysis [11].

N6-methyladenosine (m6A) methylation is involved in the initiation and development of cancers [12]. The m6A process is regulated by three types of protein, including methyltransferases (“writers”), binding proteins (“readers”), and demethylases (“erasers”). METTL3 is one of the writers and it often works with IGF2BP1, a reader protein, during m6A methylation [13–15]. The role of m6A regulators has been widely depicted in different cancers such as glioma [16], esophageal cancer [17], colorectal cancer [18], and GC [19], etc. However, its role in regulating NDUFA4 expression is unclear.

A previous study indicated that long non-coding RNA (lncRNA) MIF-AS1 could increase NDUFA4 expression to promote GC progression [10]. However, the mechanism of NDUFA4 in GC required additional investigation. Herein, our study was to comprehensively explore the pathogenic mechanism of NDUFA4 in GC.

## Materials and methods

### Bioinformatics analysis

The RNA expression data of GC were acquired from Gene Expression Omnibus (GEO) database, in which single-cell RNA-seq data were obtained from GSE134520 dataset [20] and bulk RNA-seq data were obtained from GSE33335 dataset [21]. The prognostic value of NDUFA4 in GC patients was analyzed using the GEO database provided by the Kaplan–Meier plotter (kmplot.com/analysis).

### Clinical specimens

The 30 paired GC and adjacent-normal specimens were collected from patients who received surgery at Shanghai Tongren Hospital from July 2019 to July 2021. This study was approved by the Ethics Committee of the Shanghai Tongren Hospital with written informed consent obtained. Besides, the GC tissue microarray that included 95 GC tissues and 20 normal gastric tissues was purchased from Shanghai Outdo Biotech. The inclusion criteria was defined as the certain diagnosis of GC. Also, patients who had received any treatment such as chemotherapy, radiotherapy, and biological medication (monoclonal antibodies) before the sampling were excluded from the study.

### Immunohistochemistry (IHC)

The clinical specimens in paraffin were sectioned. The anti-NDUFA4 antibody (bs-19070R; BIOSS) was applied to the section followed by the secondary antibody (D-3004, Shanghai Long Island Biotec. Co., Ltd). The expression of NDUFA4 was scored by two investigators blinded to clinical status, based on staining intensity (0, negative; 1, weak; 2, moderate; 3, strong) and percentage of positive cells (0, <5%; 1, 5–25%; 2, 25–50%; 3, 50–75%; 4, >75%). GC patients were divided into low- and high-expression groups based on the cutoff point of IHC score = 6.

### Cell culture and transfection

The human GC cell lines (AGS, HGC27, MKN28, MKN45, and MGC-803) and normal human gastric epithelium (GES-1) were obtained from Shanghai Cell Bank and authenticated by STR before shipping. Mycoplasma contamination was tested if concerned. Cells were cultured in DMEM with 10% fetal bovine serum (Gibco, Grand Island, NY, USA). siRNAs targeting METTL3 were synthesized by GenePharma. The shRNAs targeting NDUFA4 or IGF2BP1 were cloned into a pLKO.1 vector and packaged as lentivirus. The sequences of shRNA and siRNAs were listed in Supplementary Table 1.

### Cell viability

Cells were seeded in 96-well plates (3 × 10^3^/well) and incubated with 10 µl Cell Counting Kit-8 reagent for 1 h. Then the optical density at 450 nm was detected.

### Colony formation assay

Forty-eight hours after treatment, cells were seeded in 10 cm dishes and cultured for two weeks. At the end of the incubation, colonies were fixed with paraformaldehyde for 15 min and stained with 0.5% crystal violet for 30 min. Colonies with 50 cells or more were counted.

### Flow cytometry

Cells were fixed using 70% ethanol and stainedbypropidium iodide (PI) for cell cycle analysis. PI and FITC-labeled annexin V (annexin V–FITC) staining were used for cell apoptosis analysis. Beisdes, 10 µM 2′-7′dichlorofluorescin diacetate (DCFH-DA) was used to estimate reactive oxygen species (ROS) level. Moreover, mitochondrial membrane potential (MMP) ratio was calculated as red (JC-1 aggregates)/green (JC-1 monomers) fluorescence intensity using JC-1 assay kit (C2006, Beyotime Institute of Biotechnology, Jiangsu, China). Flow cytometry was conducted on CytoFLEX flow cytometry (BD Biosciences, Franklin Lakes, NJ, USA).

### Extracellular flux analysis

The level of oxygen consumption rate (OCR) and extracellular acidification rate (ECAR) was estimated using Seahorse XF24 Extracellular Flux Analyzer as previously described [22]. Briely, cells digested to a density of 1 × 10^4^/well, were seeded in XF-24 culture plates (Agilent Technologies, Santa Clara, CA, USA), and were then placed in an incubator of 37 °C and 5% CO_2_ for 24 h. Around 1 h before detection, cells were shifted into an incubator without CO_2_, and culture medium was replaced by XF Base Medium (Agilent Technologies). Subsequently, 1 μM oligomycin (ATP synthase inhibitor) was added into “A” well of Seahorse gauging plate, 1.5 μM carbonyl cyanide p-trifluoromethoxyphenylhydrazone (FCCP; uncoupler) was supplemented into “B” well and then mixture of antimycin A (complex III inhibitor; 0.5 μM) & rotenone (complex I inhibitor; 0.5 μM) was instilled into “C” well using Seahorse XF Cell Mito Stress Test Kit (Agilent Technologies). Using a Seahorse XF24 Extracellular Flux Analyzer, cellular OCR was monitored. In addition, the cells were treated sequentially with 1 μM of glucose, 1 μM of oligomycin, and 0.5 μM of 2-DG (the glycolytic inhibitor) at time points for measurement of ECAR.

### Measurement of glucose uptake

Glucose uptake was measured using a fluorescent glucose 2-NBDG (2-Deoxy-2-[(7-nitro-2,1,3-benzoxadiazol-4-yl) Glucose Uptake Assay Kit (Biovision, Milpitas, CA, USA) following manufacturer’s protocol. In brief, 5 × 10^5^ cells per well in a six-well plate were cultured at 37 °C for 24 h. At 48 h after transduction, cells were starved for glucose for 3 h. After incubating with Krebs-Ringer Bicarbonate Buffer with 2% bovine serum albumin for 40 min, 2-NBDG (100 µM) was injected into each well and incubated for 45 min at 37 °C. The cells were then washed with PBS, trypsinized, resuspended in 10% FBS before subjecting to flow cytometry analysis. The glucose uptake potential of the samples was analyzed using CytoFLEX flow cytometer (BD Biosciences).

### Measurement of lactate and ATP

The cells were seeded in 96-well plates at 3.5 × 10^3^ cells per well. After overnight incubation at 37°C, 5% CO_2_, the complete medium was changed to fresh DMEM (50 μl/well). After 24 h, the supernatant of cells was collected by centrifugation. Then, according to the manufacturer’s instructions, the lactate release was determined using Lactic Acid assay kit (Nanjing Jiancheng Bioengineering Institute, China). ATP content was measured with the ATP assay kit (Nanjing Jiancheng Bioengineering Institute, China), as per the manufacturer’s protocol. In brief, cells were seeded in the 6-well plate for 12–24 h. Then cells were harvested by using 200–300 μl lysis buffer and vortexed for 1 min. The supernatant was mixed with detection solution and then analysis for ATP concentration was normalized to the corresponding total protein amounts from each sample.

### Quantitative RT-PCR (qRT-PCR)

Total RNA was extracted using Trizolreagents. The qRT-PCR was performed using SYBR® Green kit on QuantStudio 5 system (ABI, USA). Gene expression was normalized to β-actin. The primer sequences were listed as follows: NDUFA4-F: 5ʹ-AGTCCGTAGTGTCTCATTG-3ʹ, NDUFA4-R: 5ʹ-TACAGTGTTGCTCCAGTAG-3ʹ; Cyclin D1-F: 5ʹ-AGCTCCTGTGCTGCGAAGTGGAAAC-3ʹ, Cyclin D1-R: 5ʹ-AGTGTTCAATGAAATCGTGCGGGGT-3ʹ; CDK4-F: 5ʹ-GCCTGGCCAGAATCTACAGCTAC-3ʹ, CDK4-R: 5ʹ-GGTCGGCTTCAGAGTTTCCAC-3ʹ; β-actin-F: 5ʹ-TGGCATCCACGAAACTAC-3ʹ, β-actin-R: 5ʹ-CTTGATCTTCATGGTGCTG-3ʹ.

### Western blot

The total protein was extracted by RIPA buffer, separated by SDS-PAGE, and transferred to PVDF membrane. Then it was blocked and incubated with antibodies against NDUFA4 (ab129752), OPA1 (ab157457), p-Drp1 (ab154879), PGC1α (ab106814), ENO1 (ab227978), LDHA (ab101562), METTL3 (ab195352), IGF2BP1 (ab184305), Cyclin D1 (ab16663), CDK4 (ab108357) and β-actin (ab8226, all from Abcam). The protein content was detected by enhanced chemiluminescence system (Bio-Rad, USA).

### Analysis of m^6^A content

Trizol reagent was used to extract the total RNA. Poly(A)^+^ RNA was purified using GenElute^TM^ mRNA Miniprep Kit (MRN10, Sigma, Louis, MO, USA). The m^6^A content was assayed using the m^6^A RNA Methylation Assay Kit (ab185912, Abcam). Briefly, 80 µl of binding solution and 200 ng of sample RNA were added into each designated well, and then incubated at 37 °C for 90 min for RNA binding. Wash each well three times with wash buffer. 50 µl of the diluted capture antibody was added into each well, and then incubated at room temperature for 60 min. Each well was incubated with detection antibody and enhancer solution at room temperature for 30 min subsequently. Finally, the wells were incubated with developer solution in the dark for 1 to 10 min at 25 °C. The reaction was stopped with stop solution and determined using a microplate reader at 450 nm wavelength within 2 to 10 min.

### RNA immunoprecipitation (RIP) assays

Magna RIP RNA-Binding Protein Immunoprecipitation kit (Millipore Sigma, Burlington, MA, USA) was used for the RIP assay following the manufacturer’s instructions. Cells were prepared using RIP lysis buffer and the RNA-protein complexes were incubated with anti-m6A (ab208577), anti-IGF2BP1 (ab184305) or anti-IgG antibody (ab172730, all from Abcam) overnight at 4 °C and washed with RIP-wash buffer for 10 min at 4 °C and then RIP-lysis buffer for 5 min at 4 °C. The co-precipitated RNAs were purified using phenol:chloroform:isoamyl alcohol and subjected to qRT-PCR.

### Dual-luciferase reporter assay

The NDUFA4 3′UTR sequence was cloned into the pGl3 vector (Promega, Madison, WI, USA). GC cells were tranfected with METTL3 siRNA and the pGl3-NDUFA4 3′UTR luciferase reporter plasmid and the pRL-TK vector (Promega) expressing the Renilla luciferase) using Lipofectamine 2000 (Invitrogen). A dual-luciferase assay was conducted based on the manufacturer’s protocol. The firefly luciferase activity was normalized to the Renilla luciferase activity.

### Estimation of mRNA stability

Cells were treated with 0.2 mM actinomycin D (GlpBio, Montclair, CA, USA) for 0, 3, and 6 h. The samples were then collected for total RNA extraction and cDNA synthesis, which were performed according to the methods described above. qRT-PCR was performed for the quantification of mRNA levels.

### In vivo tumor xenograft model

All animal experiments were performed under the animal ethics guidelines of Shanghai Tongren Hospital. A total of 5×10^7^ AGS cells transduced with or without NDUFA4 shRNA or MKN45 cells transduced with or without NDUFA4 overexpression lentivirus were subcutaneously injected into nude mice (4–6 weeks) (*n* = 6 each group). The 2-DG (100 mg/kg/day) and mitochondrial fission inhibitor Mdivi-1 (25 mg/kg/day) were administrated after 12 days of injection. Tumor size was detected every 3 days. The xenografts were collected after 33 days.

### Statistical analysis

Data analyses and visualization were conducted using GraphPad Prism 8.4.2. For difference comparison, the data followed a normal distribution, as confirmed by Shapiro-Wilk test. Thus, the student t-test and one-way ANOVA were used. Kaplan-Meier was used for survival analysis. *P* < 0.05 was statistically significant.

## Results

### NDUFA4 was increased in GC and its elevation indicated unfavorable prognosis

**Fig. 1:**
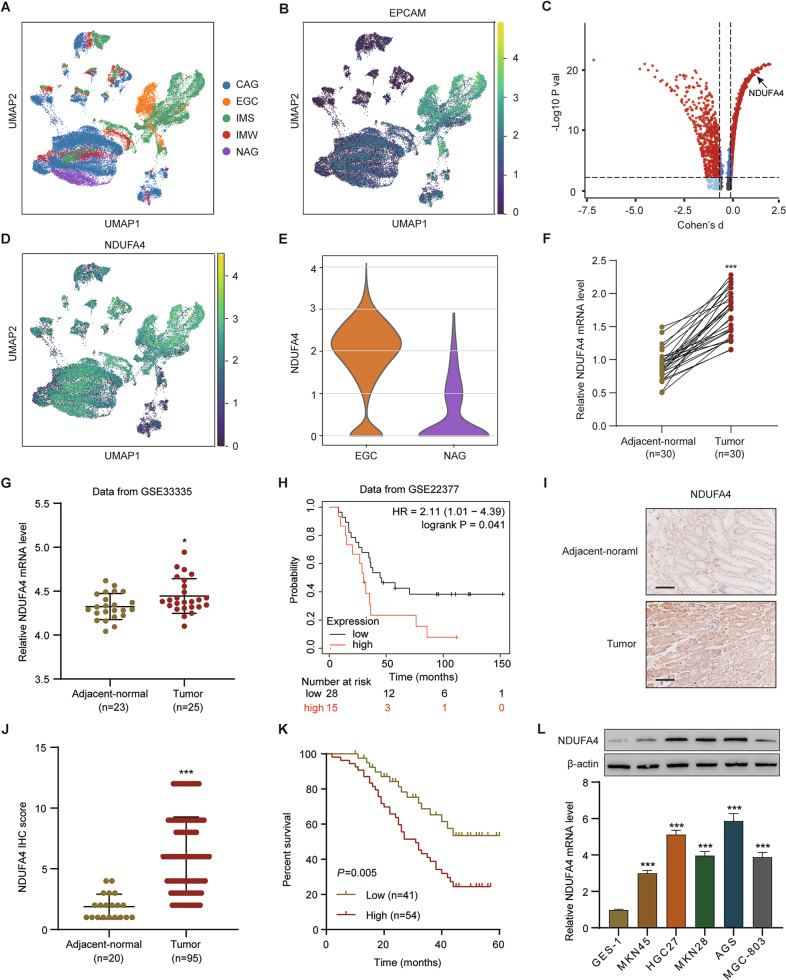
NDUFA4 was up-regulated in GC and its elevation indicated poor prognosis. **A** Uniform Manifold Approximation and Projection (UMAP) plot of patients with non-atrophic gastritis (NAG), chronic atrophic gastritis (CAG), wild intestinal metaplasia (IMW), severe intestinal metaplasia (IMS) and EGC in GSE134520 are denoted by distinct colors. **B** UMAP plot showed the distribution of epithelial cells in patients with NAG, CAG, IMW, IMS and EGC, marked by the expression of marker gene epithelial cell adhesion molecule (EPCAM) (yellow, high; purple, low). **C** Differentially expressed genes in epithelial cells between patients with EGC and NAG (the significantly expressed gene, NDUFA4, is highlighted). **D** UMAP plot of NDUFA4 expression in patients with NAG, CAG, IMW, IMS and EGC (yellow, high; purple, low). **E** NDUFA4 expressionin epithelial cells between patients with EGC and NAG. **F**, **G** NDUFA4 expression in paired gastric and adjacent normal tissues in hospital cohort (**F**) and GSE33335 dataset (**G**). **H** Survival analysis and comparison among people with high and low values of NDUFA4 expression in GSE22377 database. **I** Representative IHC images and (**J**) scores of NDUFA4 in GC tissue microarrays. Scale bar: 100 μm. **K** Survival analysis and comparison among people with high and low expression of NDUFA4 expression in GC tissue microarrays. **L** The relative mRNA and protein levels of NDUFA4 in various GC cells and normal human gastric epithelium. *P < 0.05, ***P < 0.001 vs adjacent-normal or GES-1.

The single-cell RNA-seq data of patients with gastric premalignant lesions and early gastric cancer (EGC) were downloaded from the GEO database with accession number GSE134520. This data set consists of 56,440 cells from patients with non-atrophic gastritis (NAG), chronic atrophic gastritis (CAG), wild intestinal metaplasia (IMW), severe intestinal metaplasia (IMS) and EGC, which arestructures by Uniform Manifold Approximation and Projection (UMAP) and denoted by distinct colors (Fig. 1A). UMAP plot showed the distribution of epithelial cells in patients with NAG, CAG, IMW, IMS and EGC, marked by the expression of marker gene epithelial cell adhesion molecule (EPCAM) (Fig. 1B). To determine whether the genes exert differential effects on GC tumorigenesis, we compared the single cell RNA-seq transcriptome of each epithelial cell between patients with EGC and NAG, and differentially expressed transcripts were shown in Fig. 1C, among which NDUFA4 expression in patients with NAG, CAG, IMW, IMS and EGC was also analyzed by UMAP (Fig. 1D). Moreover, NDUFA4 expression in epithelial cells from patients with EGC was higher than that with NAG (Fig. 1E). In our 30 paired GC and normal specimens, NDUFA4 was significantly elevated in GC specimens (Fig. 1F). Similarly, NDUFA4 had high expression in GC tissues than adjacent normal tissues in GSE33335 dataset (Fig. 1G). Moreover, the low expression NDUFA4 was associated with the favorable prognosis in GC patientsin GSE22377 dataset (Fig. 1H). In GC tissue microarray, the protein expression of NDUFA4 was also elevated in GC specimens, and its high expression indicated poor prognosis (Fig. 1I–K). Furthermore, the expression of NDUFA4 was notably correlated with two of the clinicopathologic characteristics, tumor size and TNM stage, in the patients with GC (Supplementary Table 2). Compared with human normal gastric epithelium, the expression of NDUFA4 were significantly elevated in GC cells especially in AGS and HGC27 (Fig. 1L). Therefore, NDUFA4 was highly expressed in GC and indicated poor prognosis in GC patients.

### NDUFA4 promoted cell proliferation and cell cycle and reduced apoptosis in GC cells

**Fig. 2:**
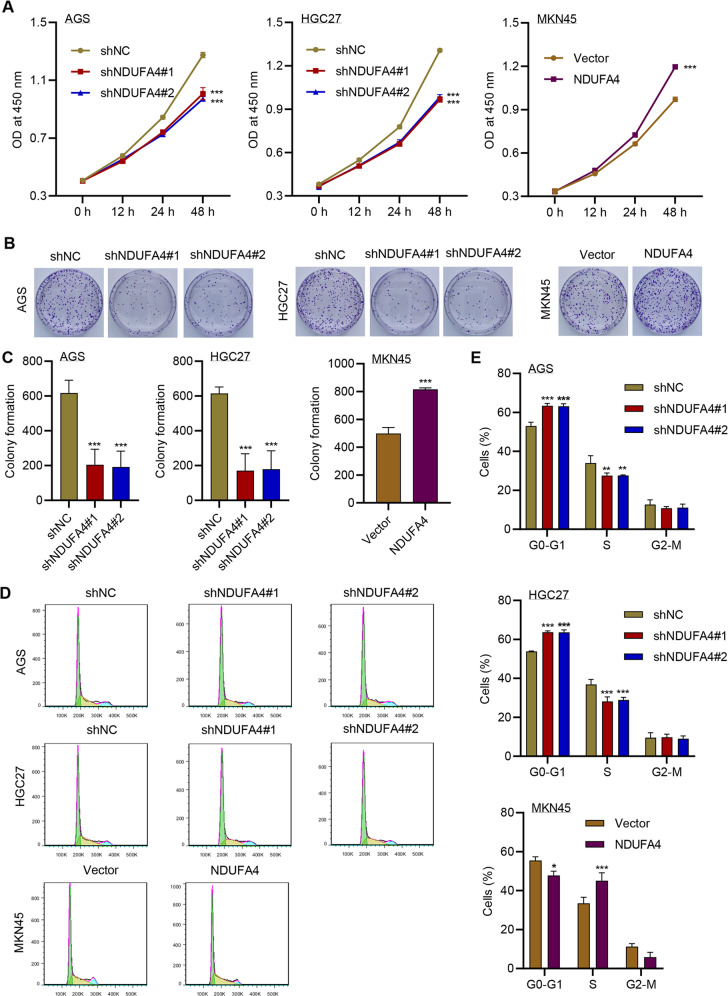
NDUFA4 promoted cell proliferation and reduced cell cycle arrest and apoptosis in GC cells. **A** Cell viability, (**B**, **C**) colony formation and (**D**, **E**) cell cycleof AGS, HGC27 and MKN45 cells with or without NDUFA4 overexpression or knockdown. *P < 0.05, **P < 0.01, ***P < 0.001 vs shNC or vector.

Then we explored the functions of NDUFA4 in GC cells. Since NDUFA4 had high expression in AGS and HGC27, and its expression was relatively low in MKN45 cells, we transduced NDUFA4 shRNA in AGS and HGC27 cells (Supplementary Fig. 1A), and overexpression lentivirus in MKN45 cells (Supplementary Fig. 1A). The knockdown of NDUFA4 significantly reduced the viability of AGS and HGC27 cells, whereas its overexpression significantly promoted the cell viability in MKN45 cells (Fig. 2A). Similarly, the colony formation was significantly inhibited by shNDUFA4 but promoted by overexpressed NDUFA4 (Fig. 2B, C). Flow cytometry revealed that the proportion of GC cells arrested at G0-G1 phase was significantly promoted by the knockdown of NDUFA4 and inhibited by overexpressed NDUFA4 (Fig. 2D, E). Western blot analysis showed that the expression of Cyclin D1 and CDK4 was inhibited by knockdown of NDUFA4 and promoted by overexpressed NDUFA4 (Supplementary Fig. 1B). Pearson correlation analysis also demonstrated the positive correlation between NDUFA4 and Cyclin D1 or CDK4 in patients with GC in the hospital cohort (Supplementary Fig. 1C, D). Moreover, cell apoptosis was significantly promoted by the knockdown of NDUFA4 in AGS and HGC27 cells and inhibited by overexpressed NDUFA4 in MKN45 cells (Supplementary Fig. 2A–C). These findings suggested that NDUFA4 could promote proliferation and cell cycle and inhibit apoptosis in GC cells.

### NDUFA4 promoted GC growth in vivo

**Fig. 3:**
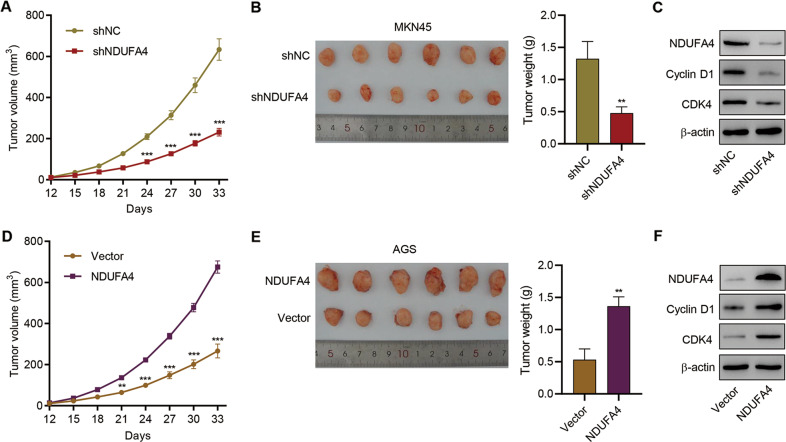
NDUFA4 promoted GC growth in vivo. AGS cells transduced with shNC or NDUFA4 shRNA vector and MKN45 cells transduced with NDUFA4 expression vector were subcutaneously injected into the armpits of the nude mice, respectively. **A**, **D** Tumor volume in each group. **B**, **E** Tumor weight in each group. **C**, **F** Expression of NDUFA4, Cyclin D1 and CDK4 in each group. ***P < 0.001 vs. shNC or vector.

Further, we verified the oncogenic role of NDUFA4 in mice. AGS cells transduced with shNDUFA4 or shNC and MKN45 cells transduced with NDUFA4 overexpression vector or blank vector were subcutaneously injected in mice. After the sacrifice of mice, we found that the tumor volume and tumor weight in shNDUFA4 group was significantly lower than shNC group (Fig. 3A, B). Western blot analysis showed that the expression of Cyclin D1 and CDK4 in tumor xenograft was inhibited by knockdown of NDUFA4 (Fig. 3C). In contrast, the overexpression of NDUFA4 significantly promoted tumor growth and expression of Cyclin D1 and CDK4 in tumor xenograft (Fig. 3D–F). These results demonstrated that NDUFA4 could promote tumor growth in GC mice model.

### NDUFA4 promotes glycolytic and oxidative metabolism in GC cells

**Fig. 4:**
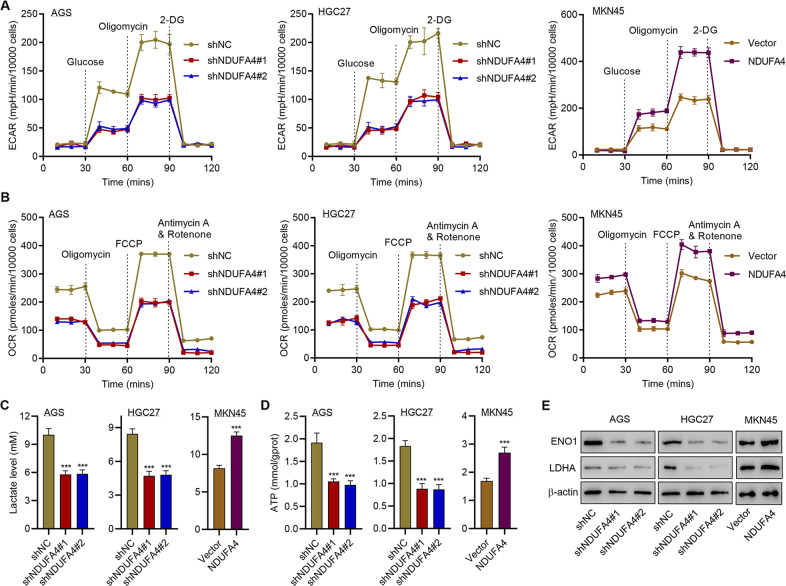
NDUFA4 promotes glycolytic and oxidative metabolism in GC cells. **A** ECAR, (**B**) OCR, (**C**) lactate, (**D**) ATP content and (**E**) expression of ENO1 and LDHA in AGS, HGC27 and MKN45 cells with or without NDUFA4 overexpression or knockdown. ***P < 0.001 vs shNC or vector.

Given that cell survival, proliferation and function are an energy-demanding process [23], we subsequently explored the potential effects of NDUFA4 on glycolytic and oxidative metabolism in GC cells. Glucose uptake was significantly inhibited by the knockdown of NDUFA4 in AGS and HGC27 cells and promoted by overexpressed NDUFA4 in MKN45 cells (Supplementary Fig. 3A, B). The knockdown of NDUFA4 significantly diminished the ECAR and OCR of AGS and HGC27 cells whereas the elevation of NDUFA4 significantly promoted ECAR and OCR in MKN45 cells (Fig. 4A, B, Supplementary Fig 4A, B). Moreover, the level of lactate and ATP was significantly reduced by the knockdown of NDUFA4 and promoted by the overexpression of NDUFA4 (Fig. 4C, D). Western blot analysis showed that the expression of glycolytic enzymes ENO1 and LDHA was significantly inhibited by the knockdown of NDUFA4 and promoted by overexpressed NDUFA4 (Fig. 4E). These findings indicated that NDUFA4 could promote glycolytic and oxidative metabolism in GC cells.

### Inhibition of glycolysis exerted anti-tumor effects against GC

**Fig. 5:**
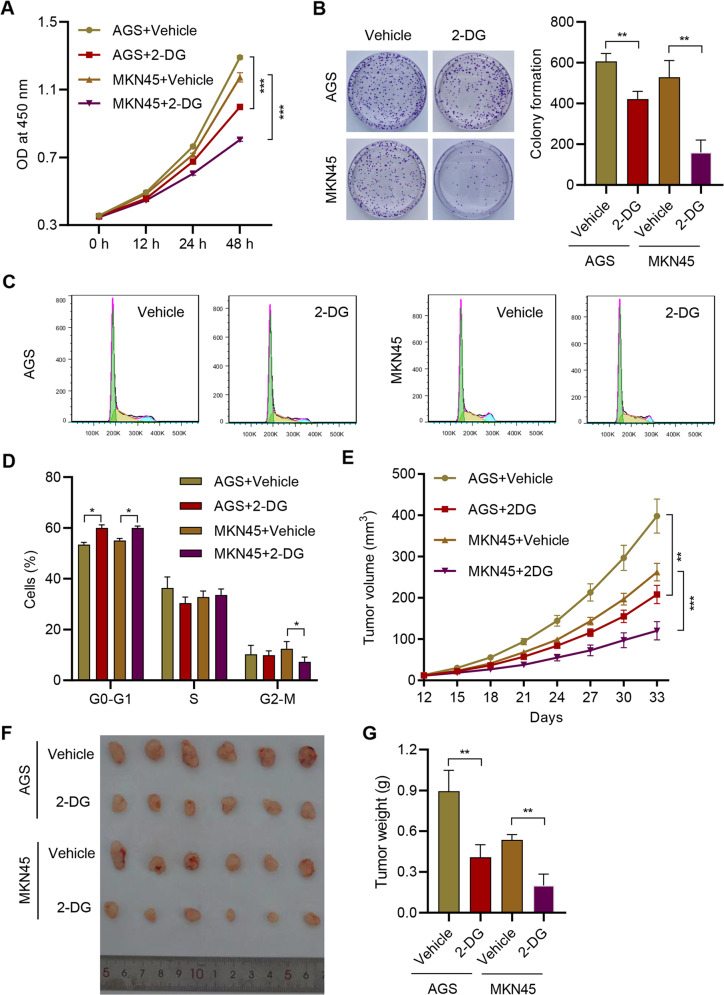
Inhibition of glycolysis exerted anti-tumor effects against GC. **A** Cell viability, (**B**) colony formation and (**C**, **D**) cell cycle of AGS and MKN45 cells treated with 10 mM 2-DG or vehicle. AGS or MKN45 cells were subcutaneously injected into the mice with or without 100 mg/kg 2-DG treatment. **E** Tumor volume was estimated. **F** The photograph of tumors. **G** Tumor weight in each group. **P < 0.01, ***P < 0.001.

Further, we employed glycolysis inhibitor, 2-DG, to explore the role of glycolysis in GC. The application of 10 mM 2-DG significantly inhibited the viability of AGS and MKN45 (Fig. 5A). The colony formation was also significantly suppressed by 2-DG (Fig. 5B). Moreover, the proportion of G0-G1 phase cells was significantly promoted by 2-DG (Fig. 5C, D). In mice, the administration of 2-DG significantly reduced the tumor growth in mice (Fig. 5E–G). These results demonstrated that the suppression of glycolysis could inhibit the proliferation and progression of GC.

### NDUFA4 regulated the level of ROS and MMP in GC cells

**Fig. 6:**
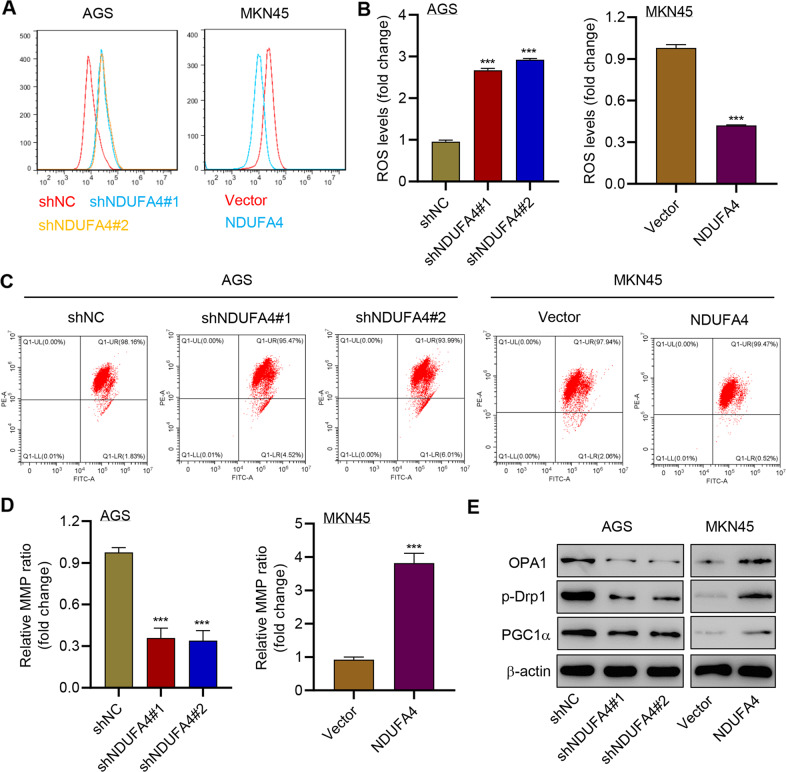
NDUFA4 regulated the level of ROS and MMP in GC cells. (**A**, **B**) ROS, (**C**, **D**) MMP and (**E**) expression of OPA1, p-Drp1 and PGC1α in AGS and MKN45 cells modulated as indicated. ***P < 0.001 vs shNC or vector.

We also explored the correlation between NDUFA4 and mitochondrial activities in GC cells. The ROS level was significantly elevated by the knockdown of NDUFA4 in AGS cells, whereas the promotion of NDUFA4 significantly reduced the ROS level in MKN45 cells (Fig. 6A, B). Besides, the knockdown of NDUFA4 significantly reduced the MMP and the overexpression of NDUFA4 exerted opposite effects (Fig. 6C, D). Meanwhile, OPA1, phosphorylated-Drp1 (p-Drp1) and PGC1α were reported to correlate with mitochondrial fusion, fission and biogenesis [24–26]. Western blot revealed that the shNDUFA4 markedly reduced the expression of OPA1, p-Drp1, and PGC1α whereas its overexpression exerted opposit effects (Fig. 6E). Therefore, these results indicated that NDUFA4 could regulate mitochondrial dynamics and biogenesis in GC cells.

### Inhibition of mitochondrial fission reversed NDUFA4-mediated effects in GC

**Fig. 7:**
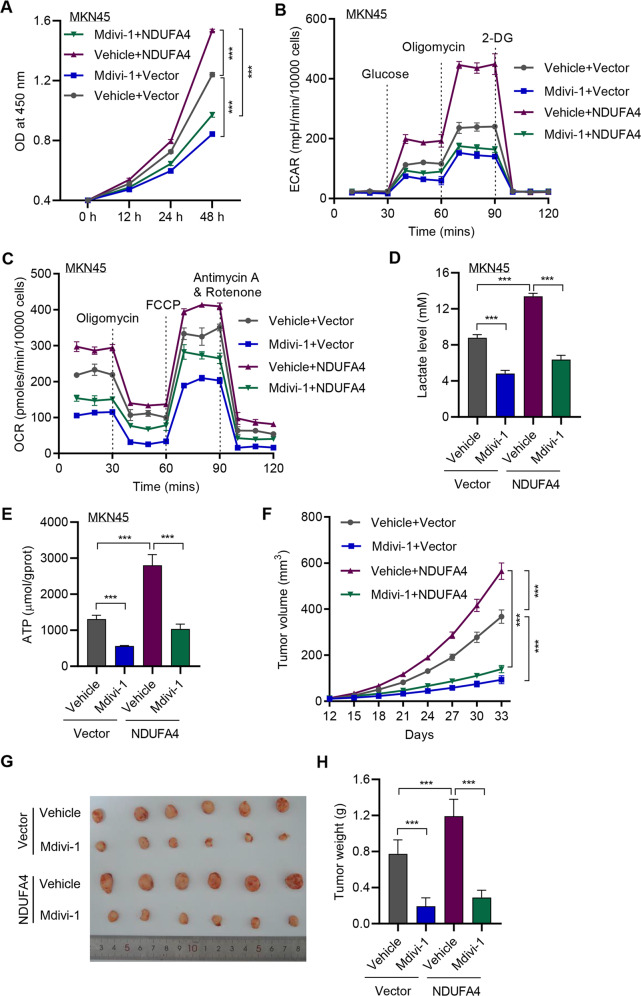
Inhibition of mitochondrial fission reversed NDUFA4-mediated effects in GC. **A** Cell viability, (**B**) ECAR, (**C**) OCR, (**D**) lactate and (**E**) ATP content of MKN45 cells with NDUFA4 overexpression and/or 20 μM Mdivi-1. MKN45 cells transduced with NDUFA4 expression vector were subcutaneously injected into the armpits of the mice with or without 25 mg/kg Mdivi-1 treatment. **F** Tumor volume in each group. **G** The photograph of xenografts. **H** Tumor weight in each group. ***P < 0.001.

Then we applied mitochondrial fission inhibitor, Mdivi-1, to verify the correlation between NDUFA4 and mitochondrial fission in GC. The application of 20 μM Mdivi-1 significantly reduced the viability of MKN45 cells, which was opposite to NDUFA4-mediated effects (Fig. 7A). Besides, the level of glucose uptake, ECAR and OCR was significantly promoted by NDUFA4 and reversed by Mdivi-1 (Fig. 7B, C, Supplementary Fig. 5A–C). Meanwhile, the production of lactate and ATP was significantly elevated by NDUFA4 but reversed by Mdivi-1 (Fig. 7D, E). In GC-bearing mice, the application of Mdivi-1 significantly reduced the tumor growth in NDUFA4 and vector groups (Fig. 7F–H). Therefore, the inhibition of mitochondrial fission could exert opposite effects against NDUFA4 in GC.

### METTL3 promoted the m6A methylation of NDUFA4 via the m6A reader IGF2BP1

**Fig. 8:**
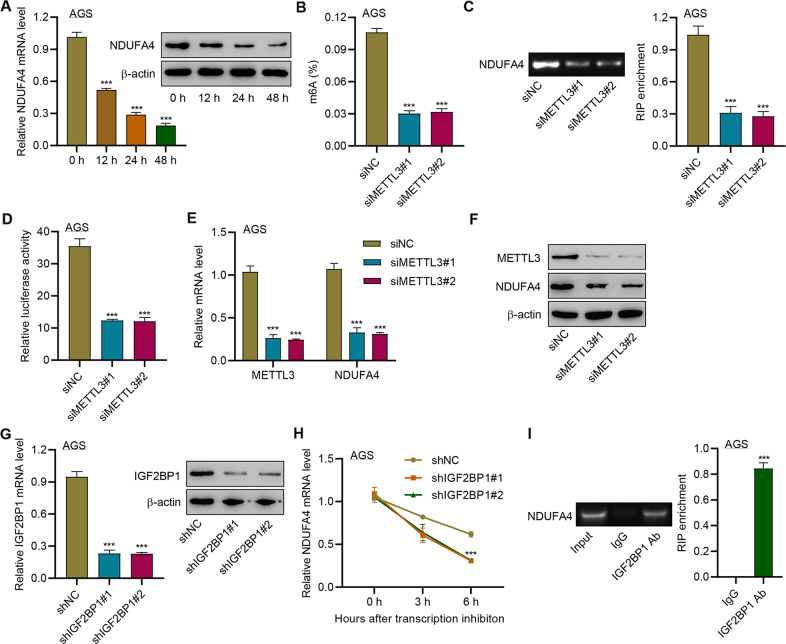
METTL3 promoted the m6A modification of NDUFA4 via the m6A reader IGF2BP1. **A** NDUFA4 expression in AGS cells treated with 100 μM 3-Deazaadenosine (DAA) for 0, 12, 24, and 48 h. **B**–**F** AGS cells were transfected with METTL14 siRNA. The whole m6A level (**B**), m6A level of NDUFA4 3’UTR (**C**), and abundance of NDUFA4 3’UTR (**D**) were detected. The expression of METTL3 (**E**) and NDUFA4 (**F**) was detected. **G**–**H** IGF2BP1 expression (**G**) and NDUFA4 mRNA stability (**H**) in AGS cells transfected with IGF2BP1 shRNA. **I** The interaction between IGF2BP1 and NDUFA4 3′UTR was detected by RIP assay. ***P < 0.001 vs 0 h, siNC, shNC or IgG.

Then we investigated the upstream regulation of NDUFA4 in GC cells. m6A modification sites of NDUFA4 were predicted by SRAMP. To further examine whether NDUFA4 expression is modulated by RNA methylation, AGS cells were treated with 3-Deazaadenosine (DAA), an inhibitor of RNA methylation. The application of 100 μM DAA significantly reduced the expression of NDUFA4 in AGS cells (Fig. 8A), which indicated the potential regulatory role of mRNA methylation in NDUFA4 expression. The inhibition of METTL3 could reduce the whole m6A level of AGS cells as well as that of the 3’UTR region of NDUFA4 mRNA (Fig. 8B, C). Moreover, the knockdown of METTL3 significantly reduced the luciferase activity of NDUFA4 mRNA (Fig. 8D). Also, it also reduced the expression of METTL3 and NDUFA4 (Fig. 8E, F). Meanwhile, we found that the transduction of shRNAs targeting IGF2BP1 could significantly reduce the expression of IGF2BP1, which led to the reduction of NDUFA4 mRNA stability (Fig. 8G, H). RIP assay demonstrated that IGF2BP1 could bind with the 3’UTR region of NDUFA4 mRNA (Fig. 8I). These results suggested that METTL3 could increase the m6A level of NDUFA4 mRNA via the m6A reader IGF2BP1 to promote the expression of NDUFA4.

## Discussion

The pathogenic mechanism underlying GC development remains to be clarified. Our study revealed that NDUFA4 was highly expressed in GC and its high expression indicated a poor prognosis. The knockdown of NDUFA4 could reduce cell proliferation and inhibit tumor growth. Meanwhile, NDUFA4 could promote glycolytic and oxidative metabolism in GC cells, whereas the inhibition of glycolysis suppressed the proliferation and tumor growth of GC. Besides, NDUFA4 inhibited ROS level and promoted MMP level in GC cells, whereas the inhibition of mitochondrial fission could reverse NDUFA4-induced glycolytic and oxidative metabolism and tumor growth of GC. Additionally, METTL3 could increase m6A level of NDUFA4 mRNA via the m6A reader IGF2BP1 to promote NDUFA4 expression in GC cells (Supplementary Fig. 6). Therefore, our study uncovered a novel pathogenic mechanism of GC progression and provided a potential target for GC treatment.

NDUFA4 is reported to correlate with the development of lung cancer [9], clear cell renal cell carcinoma [8], and colorectal cancer [11]. NDUFA4 was a biomarker for cancer-specific survival of patients with clear cell renal cell carcinoma [8]. Besides, NDUFA4 was the target of miR-7 that promoted the progression of lung cancer cells via activating AKT and ERK pathways [9]. Moreover, NDUFA4 was involved in the oncogenic mechanism of lncRNA MIFG-AS1 in colorectal cancer [11]. In GC, NDUFA4 was upregulated by lncRNA MIF-AS1, which could promote proliferation and reduce cell apoptosis [10]. This result was consistent with our findings that NDUFA4 was highly expressed in GC and correlated with the poor prognosis of GC patients. Gastric adenocarcinoma is histologically divided into intestinal, diffuse, mixed, and indeterminate subtypes, whereas the diffuse type of GC usually has a shorter duration of disease and poor prognosis [27]. Our results have revealed that NDUFA4 was highly expressed in all HER2-negative GC cell lines compared with normal human gastric epithelium, in which intestinal-type GC cell lines (MKN28, AGS) showed higher NDUFA4 expression in comparison to intestinal diffuse-type GC cell lines (MKN45). Therefore, further study is need to fully elucidate the role and expression pattern of NDUFA4 in different histological forms of GC. In GC, NDUFA4 could promote cell proliferation and tumor growth. Therefore, NDUFA4 was implicated to play an oncogenic role in GC and was a potential target for GC treatment.

Cell metabolisms such as glycolysis and mitochondrial dynamics, including fusion and fission were distinct characteristics of cancer cells [28, 29]. Several studies have shown that NDUFA4 encodes a subunit of the electron transport chain complex belonging to the respiratory chain of mitochondria to produce ATP, regulates oxidative phosphorylation pathway-related proteins such as COX6C, COX5B and NDUFA8 in GC cells [10], and promotes glucose uptake, lactate production and major enzymes involved in glycolysis such as PDK1, PFK1, PKM2 in colorectal cancer cells [11]. We demonstrated that NDUFA4 promotes glycolysis by regulating the expression of glycolytic enzymes, including ENO1 and LDHA and modulates mitochondrial dynamics and biogenesis by regulating the expression of OPA1, p-Drp1, and PGC1α. Previous studies have reported that NDUFA4 promotes the progression of lung cancer cells via activating PI3K/AKT pathway [9] and the PI3K/AKT pathway is responsible for glycolysis by regulating HIF-1α target genes ENO1 and LDHA [30, 31]. These data indicate that NDUFA4 may regulate ENO1 and LDHA expression via the PI3K/AKT/HIF-1α signaling pathway. However, whether other glycolytic enzymes are regulated by NDUFA4 should be further determined. It is possible that NDUFA4 facilitates glycolytic/oxidative metabolism by regulating these proteins. The elevation of cell glycolysis was demonstrated to promote cancer progression [32]. In addition, not only cell viability and proliferation, but also tumor metastasis and metabolism were accelerated by glycolysis and mitochondrial dynamics and biogenesis [33–37]. The lactate produced by glycolysis increased cell motility and drug resistance and its expression in tumors strongly indicated metastasis [38, 39]. In this study, we found that NDUFA4 could promote cell glycolysis and lactate production in GC, whereas the inhibition of glycolysis by 2-DG could reverse NDUFA4-mediated effects on tumor growth in vitro and in vivo. Since ROS level and MMP were essential for mitochondrial fission and they were significantly mediated by NDUFA4. Moreover, the inhibition of mitochondrial fission by Mdivi-1 could reverse NDUFA4-mediated effects on glycolytic/oxidative metabolism in vitro and tumor growth in vivo. Therefore, our results revealed that the GC progression might benefit from glycolytic/oxidative metabolism. Nevertheless, further study is need to fully validate the correlation between glycolytic/oxidative metabolism and GC progression.

METTL3 is one of the m6A writers and is involved in the development of various cancers [40]. Wang et al. revealed that the overexpression of METTL3 in GC cells resulted in the significantly promoted m6A level [41]. Besides, METTL3 was shown to promote the methylation of SEC62 mRNA that was stabilized by IGF2BP1, and the increased expression of SEC62 could promote the proliferation of GC cells [42]. However, the association between METTL3 and NDUFA4 in GC remained unclear. Herein, our study revealed that NDUFA4 expression was significantly reduced by DAA, a potent inhibitor of methyltransferase activity. METTL3 could promote the m6A level of NDUFA4 mRNA via the reader IGF2BP1. Our study revealed the upstream regulation of NDUFA4 that contributed to the progression of GC. Previous studies showed that the expression of m6A writers (METTL3, RBM15, WTAP), eraser (FTO, ALKBH5) and reader (YTHDF3, YTHDC2) was associated with pathological stage, tumor stage, andprognosis of patients with GC [43, 44]. IGF2BP2 and IGF2BP3 also regulated GC cell proliferation, migration and invasion [45, 46]. Therefore, whether other writers, erasers and readers contribute to the m6A methylation of NDUFA4 in GC cells need to be further confirmed.

To sum up, our study revealed that NDUFA4 was increased by m6A methylation and could promote GC development via enhancing cell glycolysis and mitochondrial fission. NDUFA4 was a potential target for GC treatment.

## Supplementary information

Supplementary materials

Blots

aj-checklist

## Data Availability

All data presented in this study are included within the paper and its Supplementary files.
