# Supplementary material for: m6A RNA methylation-mediated NDUFA4 promotes cell proliferation and metabolism in gastric cancer
Source: Cell Death Dis. 2022 Aug 17;13(8):715. doi: 10.1038/s41419-022-05132-w (PMC9385701; doi:10.1038/s41419-022-05132-w)
Supplement: Supplementary file 1 — Supplementary materials [file 41419_2022_5132_MOESM1_ESM.docx]

**Supplementary Table 1. The sequences of shRNA or siRNA used in the study**

| **Name** | **Sequences** |
| --- | --- |
| shNDUFA4#1 | GTGTCTCATTGCAGATAAT |
| shNDUFA4#2 | GATGTTTGTTGGGACAGAA |
| shIGF2BP1#1 | GGACTTGGAGAAAGTGTTT |
| shIGF2BP1#2 | GGCTCAGTATGGTACAGTA |
| siMETTL3#1 | GCUGCACUUCAGACGAAUUTT |
| siMETTL3#2 | GGAUACCUGCAAGUAUGUUTT |

**Supplementary Table 2.** Relationship between NDUFA4 expression and clinicopathological features of 95 patients with GC

| Clinicopathological features | NDUFA4 | | *P* value |
| --- | --- | --- | --- |
|  | Low (n=41) | High (n=54) |  |
| **Gender** |  |  | 0.309 |
| Male (n=52) | 20 | 32 |  |
| Female (n=43) | 21 | 22 |  |
| **Age (years)** |  |  | 0.131 |
| ≤60 (n=36) | 12 | 24 |  |
| >60 (n=59) | 29 | 30 |  |
| **Location** |  |  | 0.115 |
| Cardia (n=41) | 20 | 21 |  |
| Corpus (n=30) | 15 | 15 |  |
| Antrum (n=24) | 6 | 18 |  |
| **Tumor size (cm)** |  |  | 0.024 |
| ≤3 (n=50) | 27 | 23 |  |
| >3 (n=45) | 14 | 31 |  |
| **TNM stage** |  |  | 0.005 |
| I (n=25) | 16 | 9 |  |
| II (n=30) | 16 | 14 |  |
| III (n=32) | 7 | 25 |  |
| IV (n=8) | 2 | 6 |  |

Differences between groups were done by the Chi-square test.


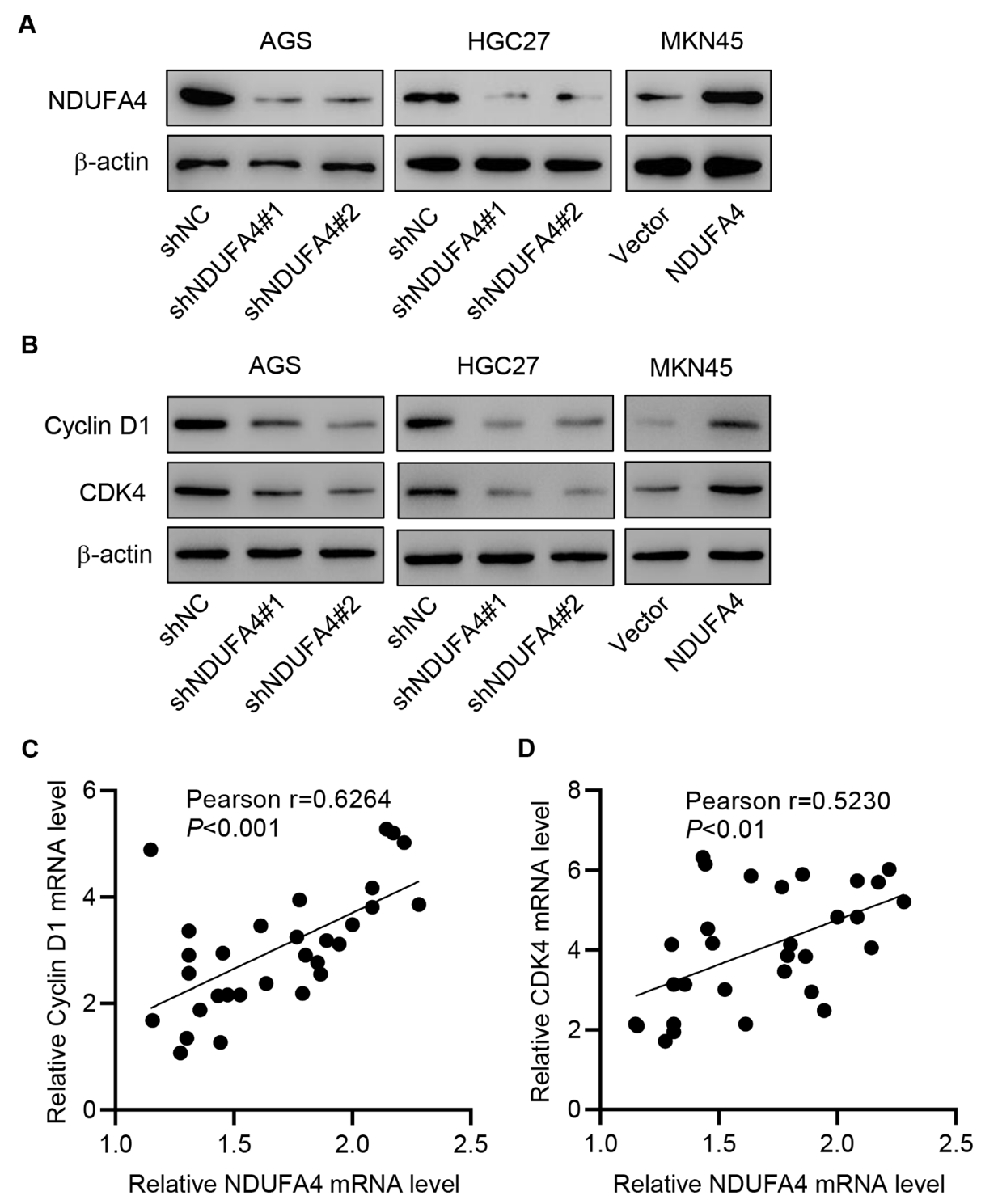


**Supplementary Figure 1.** (A, B) Expression of NDUFA4, Cyclin D1 and CDK4 in AGS, HGC27 and MKN45 cells with or without NDUFA4 overexpression or knockdown. Pearson correlation scatter plots for NDUFA4-Cyclin D1 (C) and NDUFA4-CDK4 (D) of GC patients in hospital cohort (n=30).

**
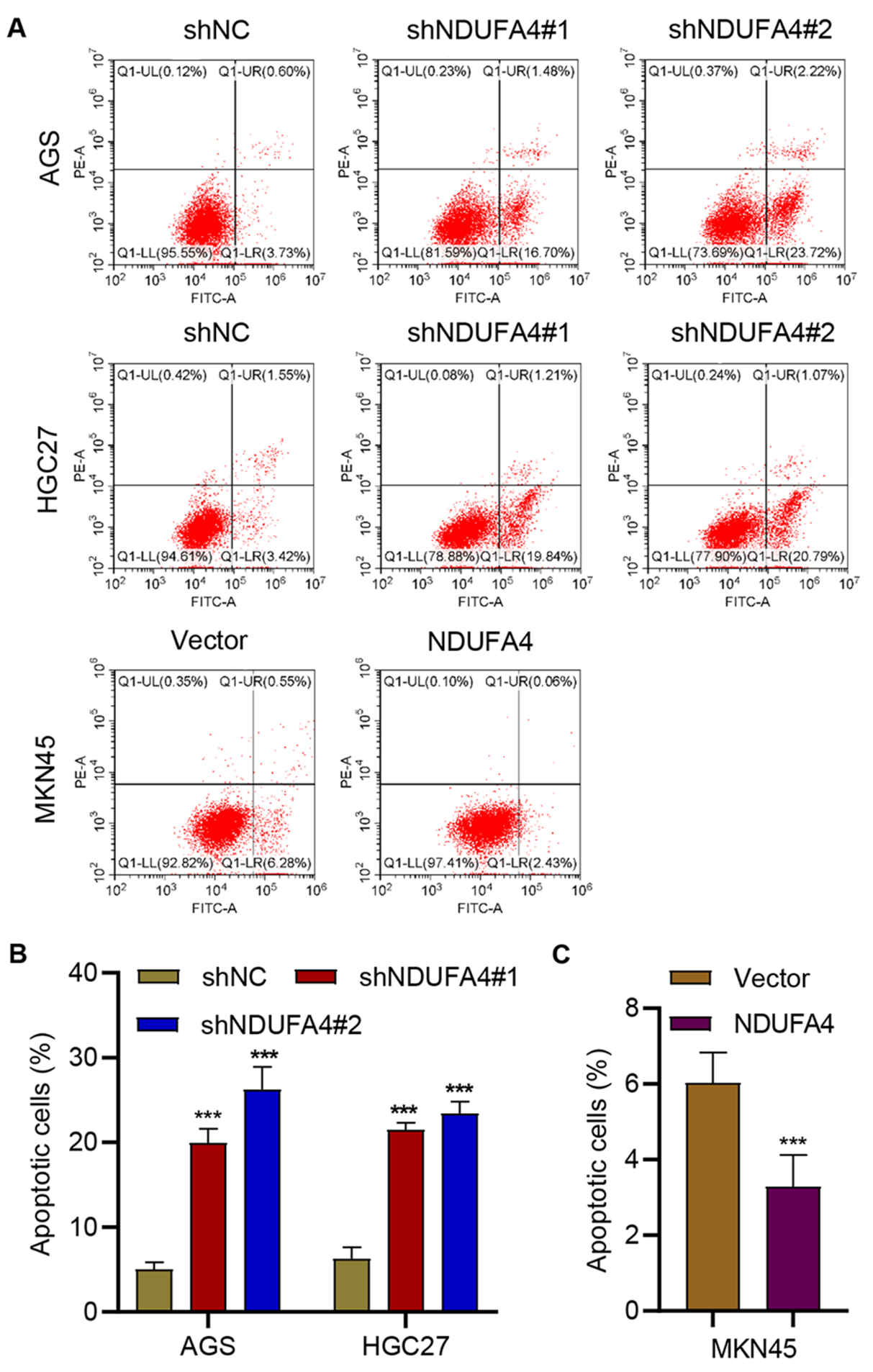
**

**Supplementary Figure 2.** (A-C) Cell apoptosis of AGS, HGC27 and MKN45 cells with or without NDUFA4 overexpression or knockdown. ****P*<0.001 vs shNC or vector.


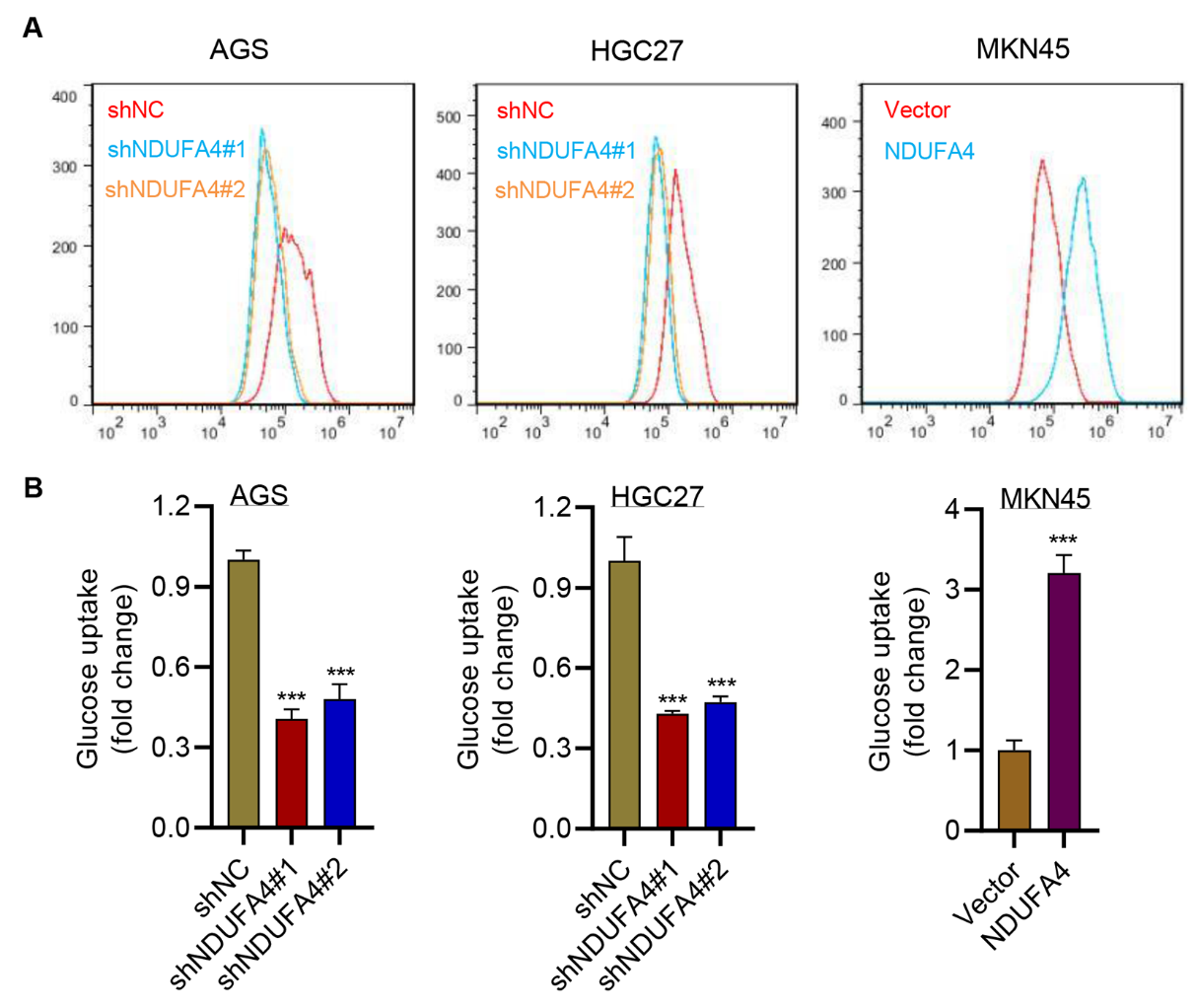


**Supplementary Figure 3.** (A-B) Glucose uptake of AGS, HGC27 and MKN45 cells with or without NDUFA4 overexpression or knockdown. ****P*<0.001 vs shNC or vector.


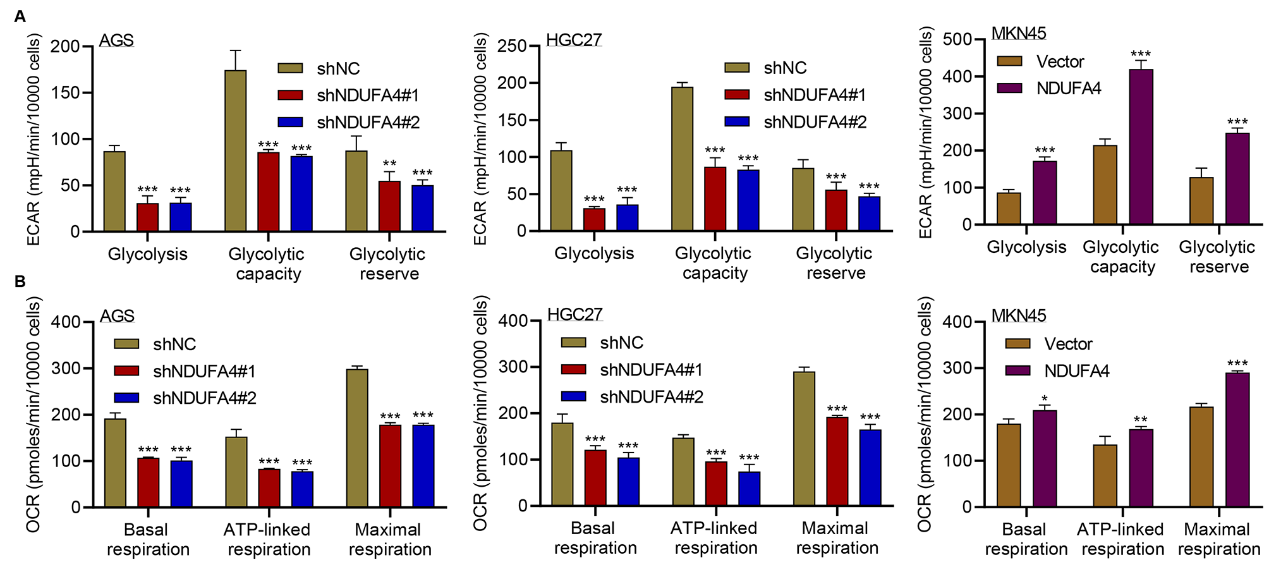


**Supplementary Figure 4.** ECAR (A) and OCR (B) of AGS, HGC27 and MKN45 cells with or without NDUFA4 overexpression or knockdown. **P*<0.05, ***P*<0.01, ****P*<0.001 vs shNC or vector.

**
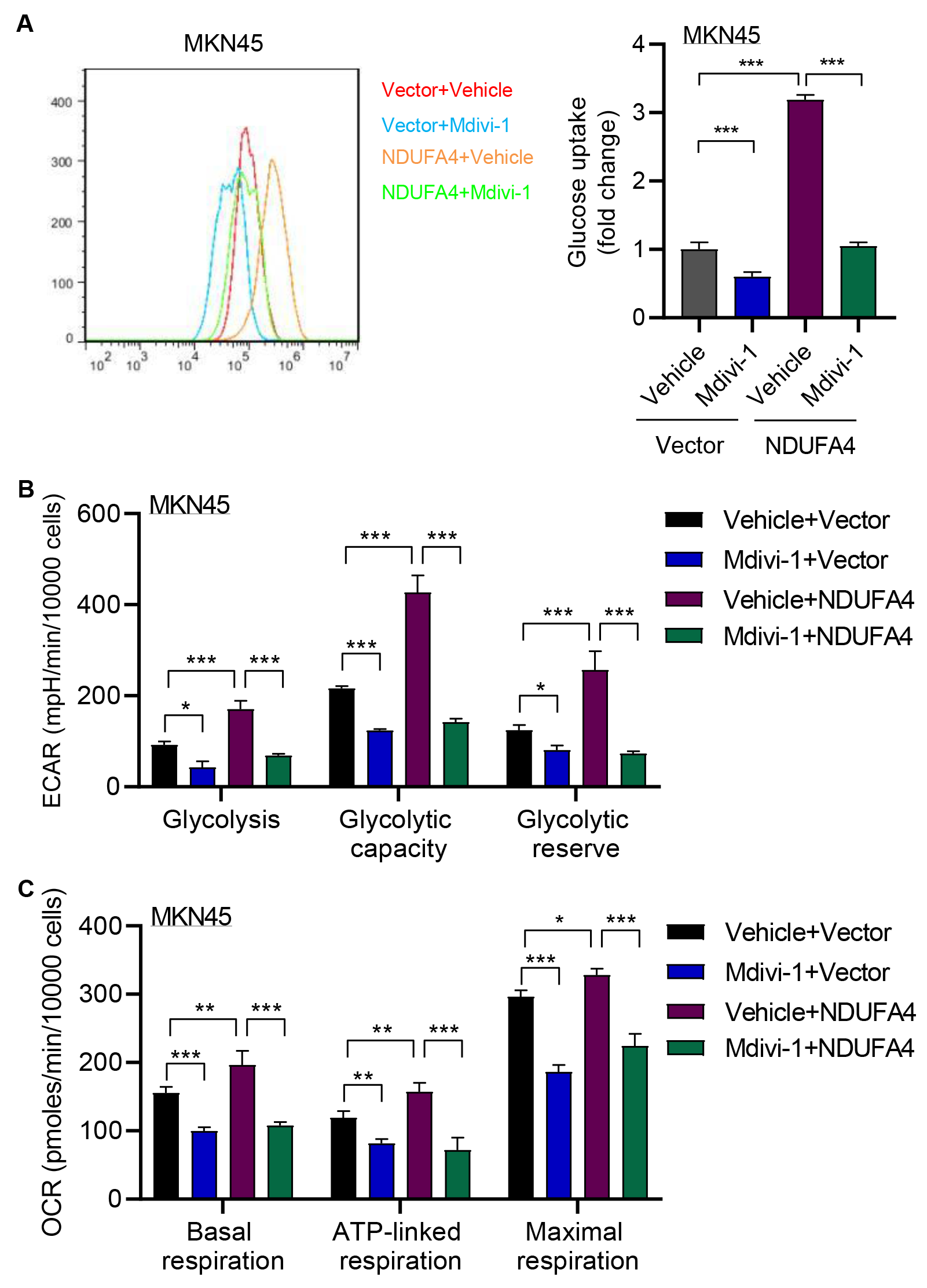
**

**Supplementary Figure 5.** Glucose uptake (A), ECAR (B), and OCR (C) of MKN45 cells with NDUFA4 overexpression and/or 20 μM Mdivi-1. **P*<0.05, ***P*<0.01, ****P*<0.001.


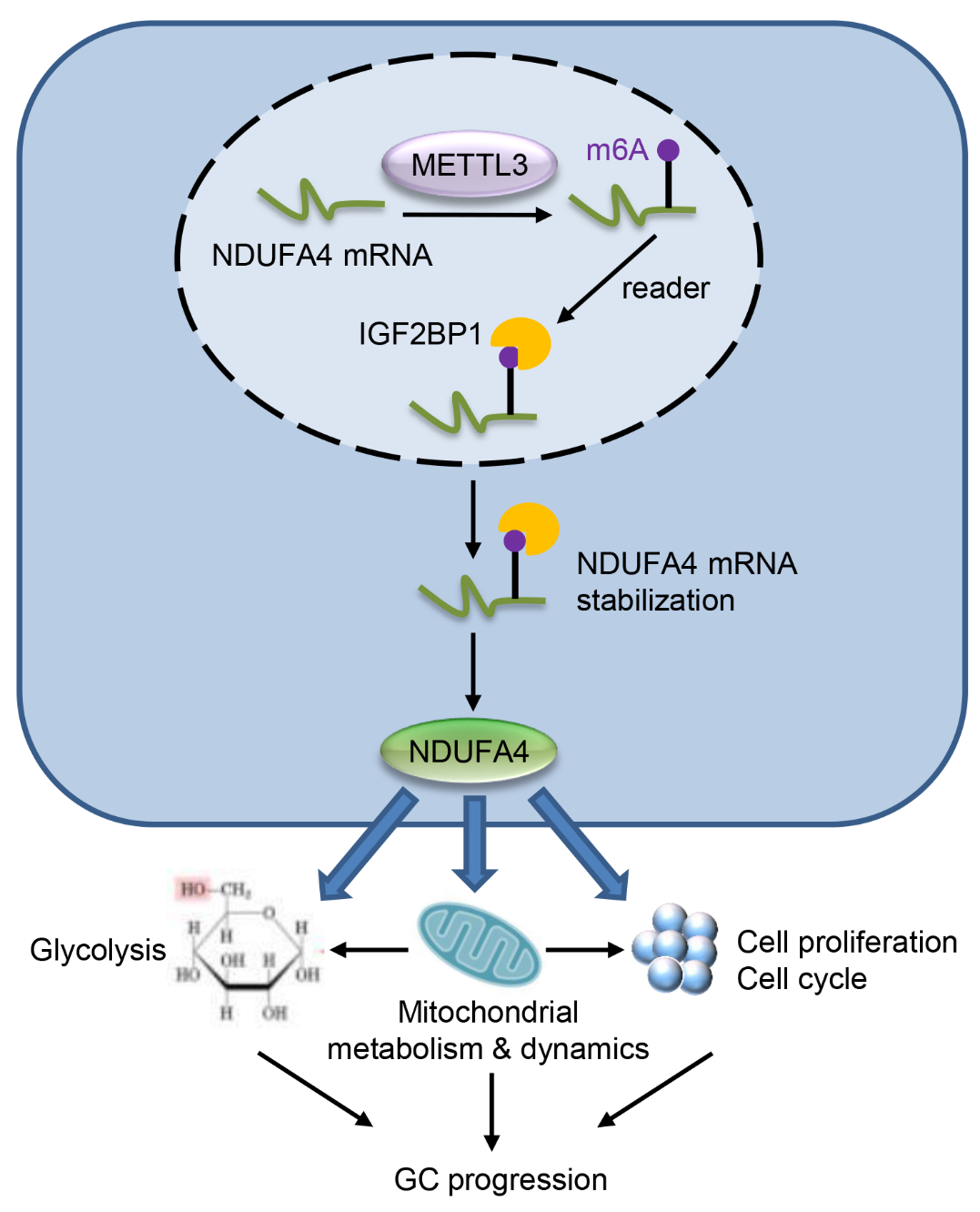


**Supplementary Figure 6.** Schematic representation of the regulation of GC cell proliferation and metabolism by NDUFA4 which is stabilized by METTL3/IGF2BP1- mediated m6A modification.
