# Supplementary material for: m6A RNA methylation-mediated NDUFA4 promotes cell proliferation and metabolism in gastric cancer
Source: Cell Death Dis. 2022 Aug 17;13(8):715. doi: 10.1038/s41419-022-05132-w (PMC9385701; doi:10.1038/s41419-022-05132-w)
Supplement: Supplementary file 2 — Blots [file 41419_2022_5132_MOESM2_ESM.pdf]

Figure 1L

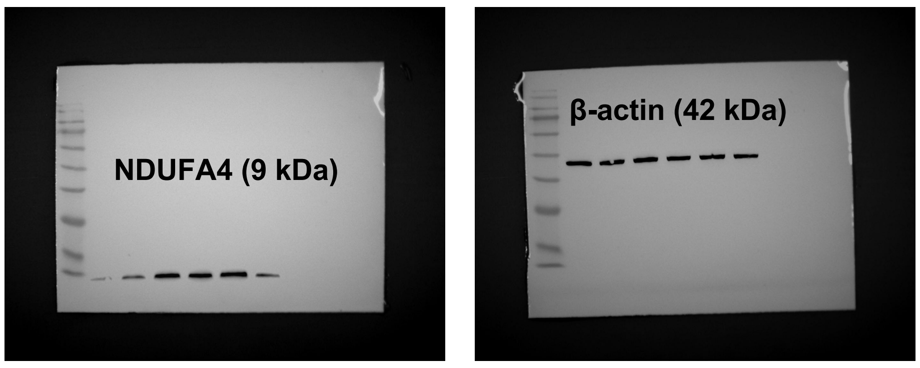

Figure 3C

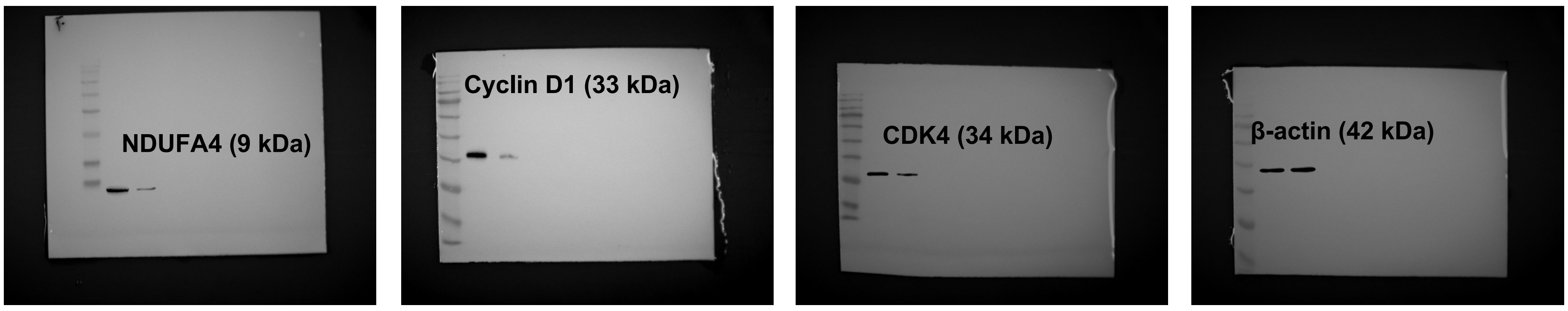

Figure 3F

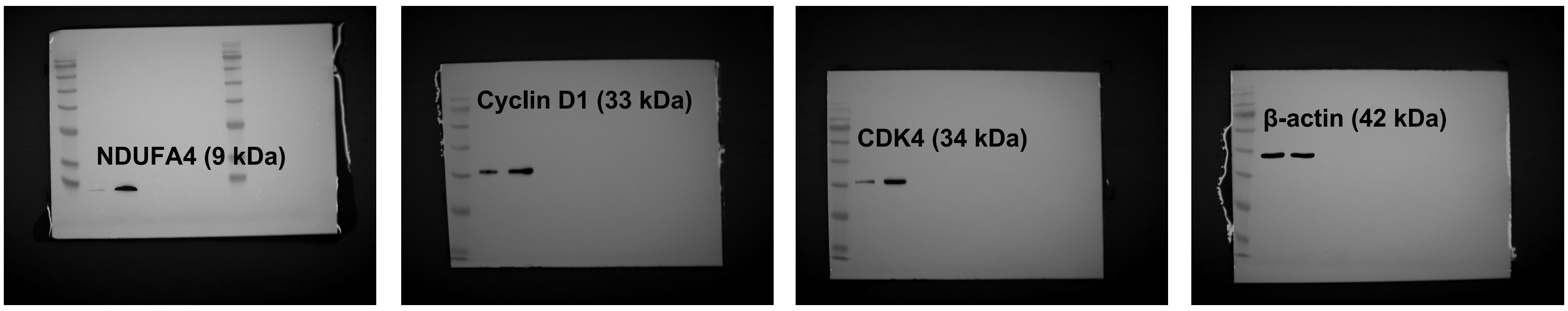

Figure 4E (AGS)

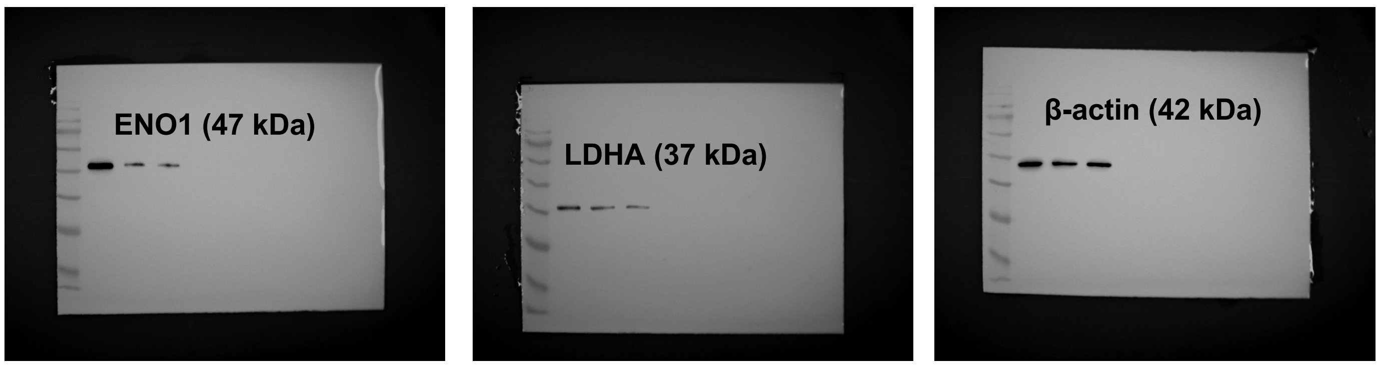

Figure 4E (HGC27)

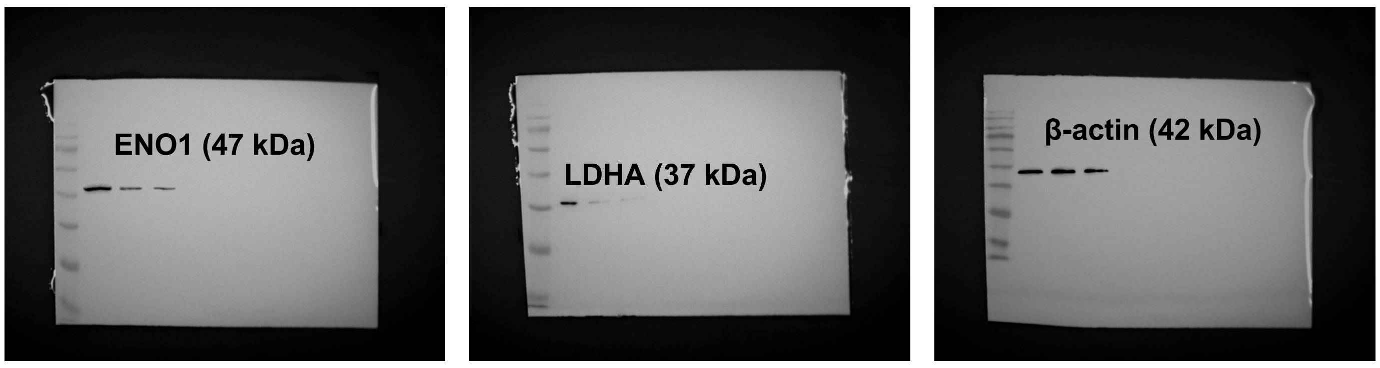

Figure 4E (MKN45)

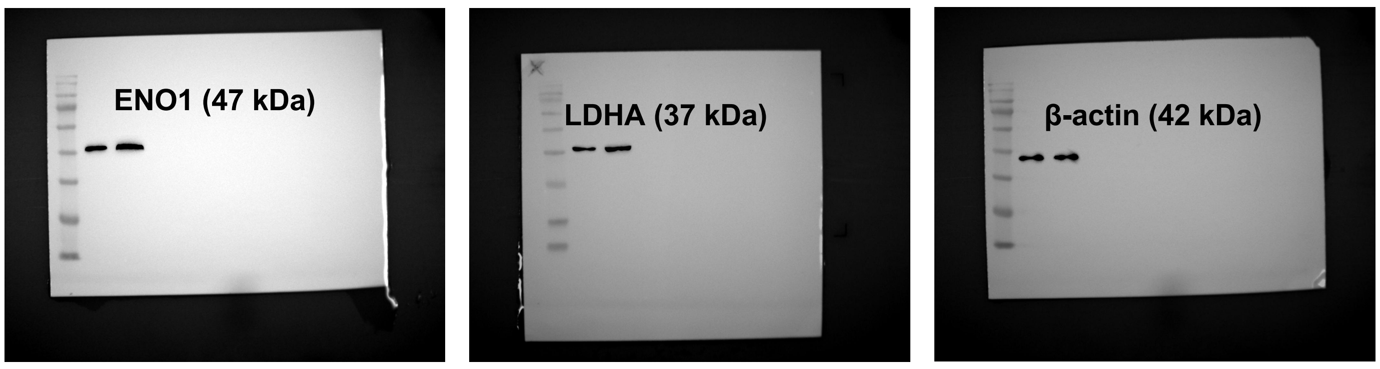

Figure 6E (AGS)

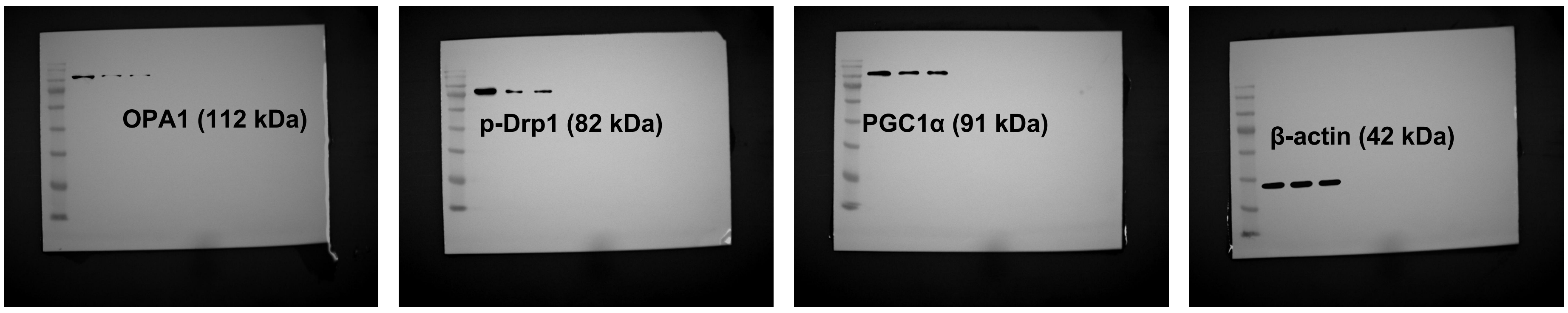

Figure 6E (MKN45)

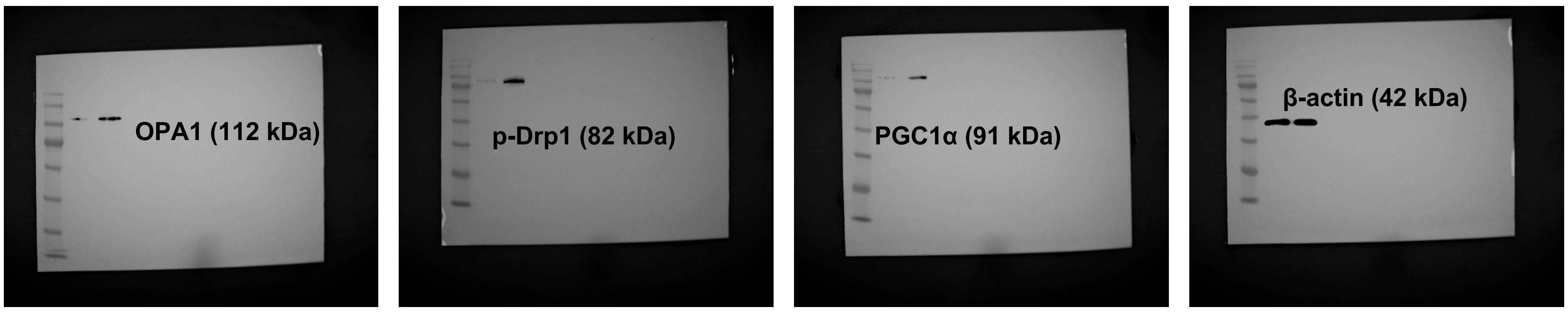

Figure 8A

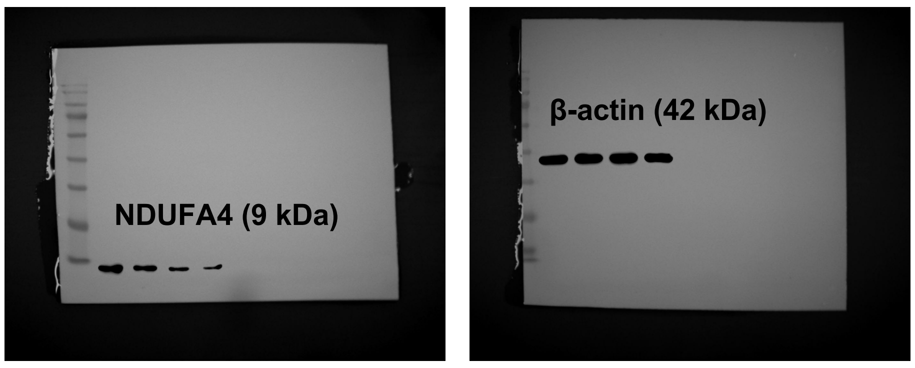

Figure 8F

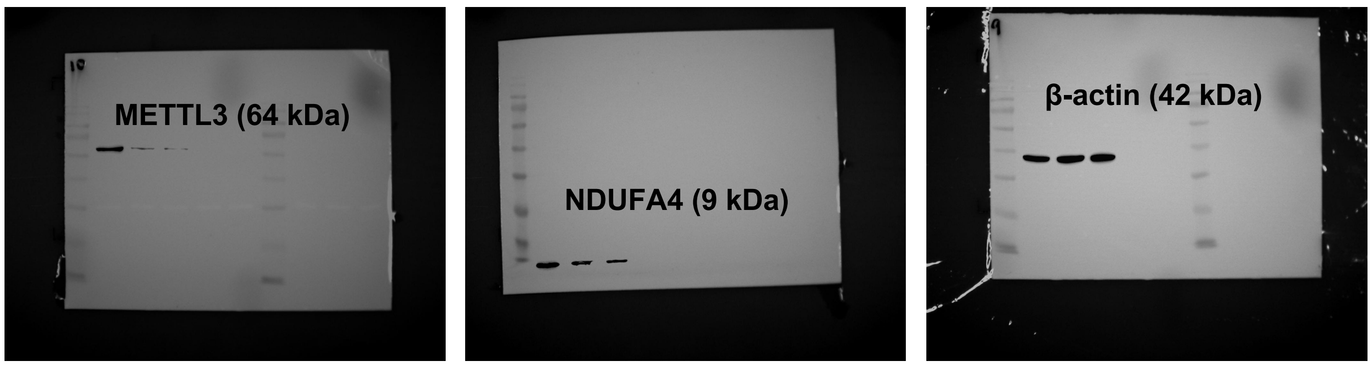

Figure 8G

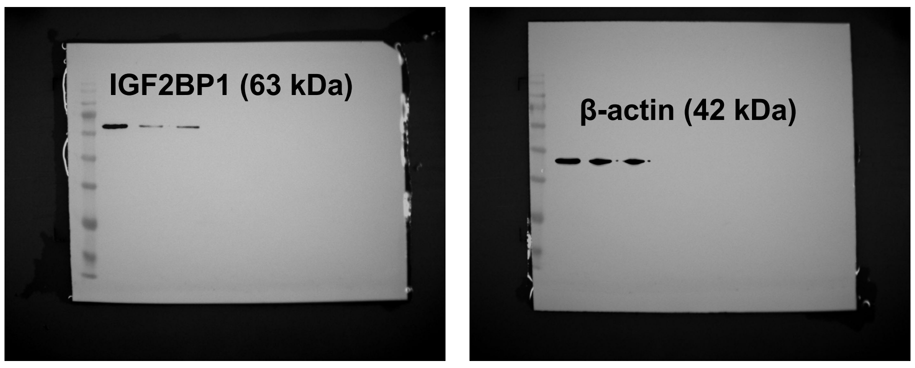

AGS

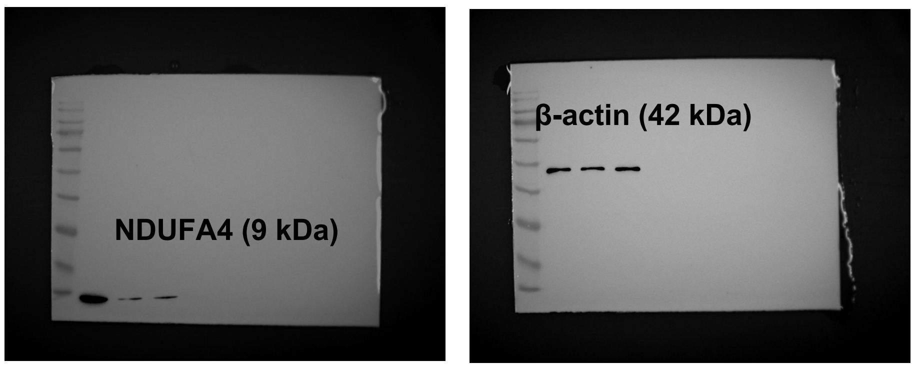

HGC27

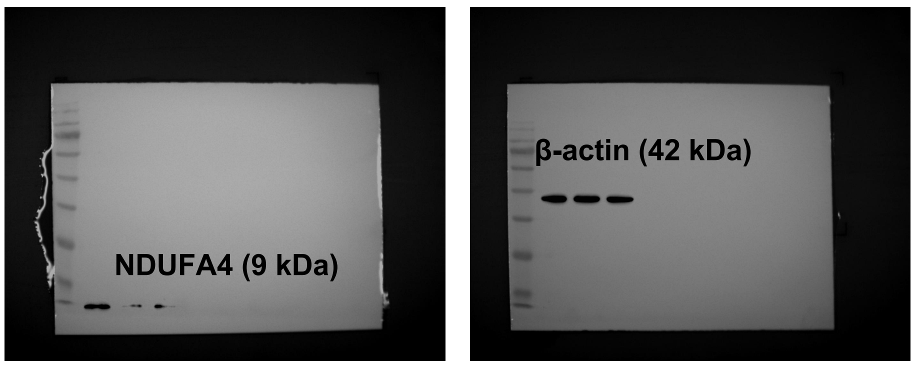

MKN45

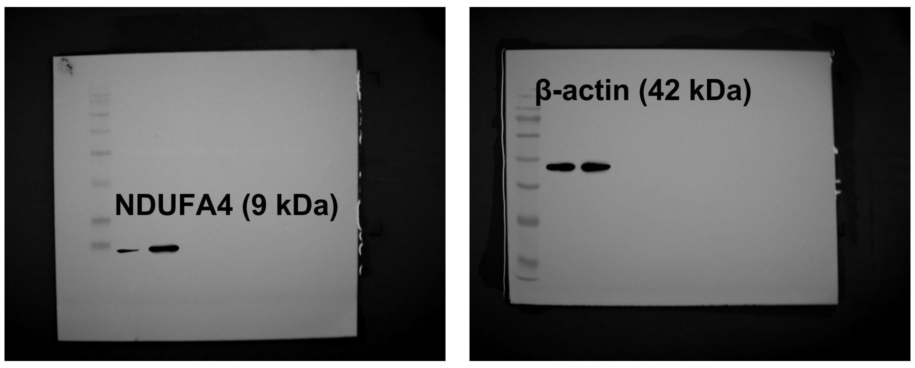

Supplementary Figure 1A

AGS

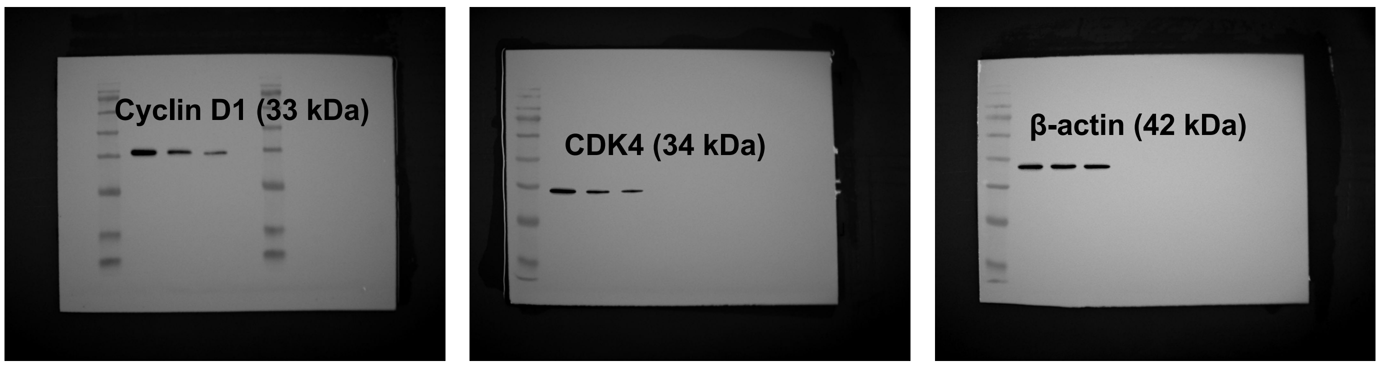

HGC27

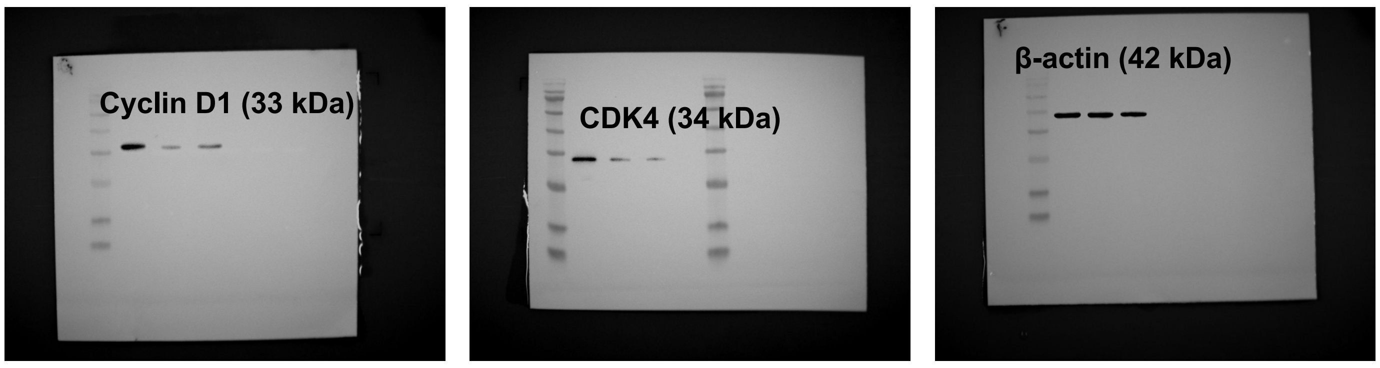

MKN45

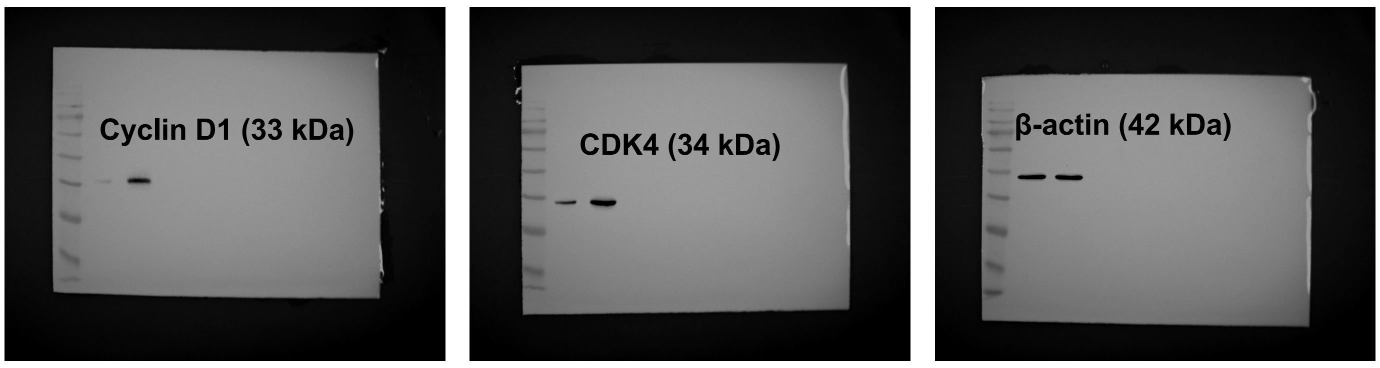

Supplementary Figure 1B
